# *Drosophila melanogaster*: A Powerful Tiny Animal Model for the Study of Metabolic Hepatic Diseases

**DOI:** 10.3389/fphys.2021.728407

**Published:** 2021-09-16

**Authors:** Karen C. M. Moraes, Jacques Montagne

**Affiliations:** ^1^Laboratório de Sinalização Celular e Expressão Gênica, Departamento de Biologia Geral e Aplicada, Instituto de Biociências, UNESP, Rio Claro, Brazil; ^2^Institute for Integrative Biology of the Cell (I2BC), CEA, CNRS, Université Paris-Saclay, Gif-sur-Yvette, France

**Keywords:** alternative animal model, *Drosophila*, genetics, liver diseases, system biology

## Abstract

Animal experimentation is limited by unethical procedures, time-consuming protocols, and high cost. Thus, the development of innovative approaches for disease treatment based on alternative models in a fast, safe, and economic manner is an important, yet challenging goal. In this paradigm, the fruit-fly *Drosophila melanogaster* has become a powerful model for biomedical research, considering its short life cycle and low-cost maintenance. In addition, biological processes are conserved and homologs of ∼75% of human disease-related genes are found in the fruit-fly. Therefore, this model has been used in innovative approaches to evaluate and validate the functional activities of candidate molecules identified via *in vitro* large-scale analyses, as putative agents to treat or reverse pathological conditions. In this context, *Drosophila* offers a powerful alternative to investigate the molecular aspects of liver diseases, since no effective therapies are available for those pathologies. Non-alcoholic fatty liver disease is the most common form of chronic hepatic dysfunctions, which may progress to the development of chronic hepatitis and ultimately to cirrhosis, thereby increasing the risk for hepatocellular carcinoma (HCC). This deleterious situation reinforces the use of the *Drosophila* model to accelerate functional research aimed at deciphering the mechanisms that sustain the disease. In this short review, we illustrate the relevance of using the fruit-fly to address aspects of liver pathologies to contribute to the biomedical area.

## Introduction

In modern societies, obesity has become a prevalent problem; according to the World Health Organization (WHO), more than 600 million adults were obese in 2016 (WGO, 2020). Consequently, metabolic dysfunctions have considerably increased, including hepatic diseases, which are one of the top 10 causes of death worldwide (Asrani et al., 2019; WHO, 2020; Powell et al., 2021). These diseases classified as acute or chronic may progress to irreversible damage and malfunction of the liver, while therapeutic strategies are still limited. To address this problem, innovative drugs and natural products have been explored; however, clinical trials have their own limitations, considering patient safety against the adverse side effects of pharmaceutical products, extensive time required for analyses, and ethical issues. Thus, to help investigating new compounds in preclinical stages, the *Drosophila melanogaster* model has become of utmost importance. *Drosophila* genetics has been used for studying many biological processes and is now a powerful alternative model to investigate metabolic changes associated with disease settings. The fruit-fly has a short life cycle, which enables analyses of the effects of drugs on various metabolic routes in a short time (Tennessen et al., 2014; Men et al., 2016; Lee et al., 2020). Furthermore, relevant biological processes and disease-related genes are conserved between humans and *Drosophila* (Pandey and Nichols, 2011; Ugur et al., 2016), despite the flies lower genomic complexity compared to that of humans. In this minireview we discuss the advantages offered by the fruit-fly model to investigate hepatic dysfunctions in a translational perspective.

## The Burden of Hepatic Diseases Needs Translational Science

The liver is a critical organ for the maintenance of body homeostasis; it controls several molecular and metabolic routes and also acts in detoxification processes (Ramos et al., 2021). In metabolic disorders, lipids may accumulate in the hepatocytes contributing to the establishment of fatty liver, also known as hepatic steatosis. This clinical condition, which happens when triacylglycerols (TAGs) represent at least 5% of the liver weight (Ikura, 2014; Powell et al., 2021; Ramos et al., 2021), can remain asymptomatic for several years or progress to more deleterious stages. Non-alcoholic fatty liver disease (NAFLD) is one of the most common forms of liver pathologies affecting ∼25% of the world population (Younossi et al., 2018). In addition, drug abuse, environmental contaminants, imbalanced diet, and viral infections may also contribute to the development of NAFLDs (Byrne and Targher, 2015). More dramatically, NAFLD favors the development of chronic hepatitis that potentially progresses to cirrhosis, increasing the risk for HCC (Friedman et al., 2018; Powell et al., 2021). The worldwide panel of liver pathologies also comprises chronic viral hepatitis, alcoholic liver disease, metabolic and cholestatic liver dysfunctions (Asrani et al., 2019; WGO, 2020). Although NAFLD was identified more than 50 years ago (Parise, 2019; Sanyal, 2019), the therapeutic options to treat or at least to control these diseases rely on long-term procedures and are still limited to weight loss and diet modification, stressing the critical need for innovative strategies based on fast and reliable preclinical tests.

Translational science aims at using the scientific discoveries from preclinical models to set up the bases for clinical trials (Gilliland et al., 2016; van Erk et al., 2021). Knowledge on liver pathologies has now increased, leading to the characterization of the biochemical mechanisms underlying disease progression and to the identification of specific biomarkers (Friedman et al., 2018; Samuel and Shulman, 2018; Eslam and George, 2020). Advances in our understanding of lipid and sugar metabolism along with omics studies and mechanistic investigations, have revealed important regulatory functions for the transcription factors sterol-regulatory-element-binding-proteins (SREBPs), carbohydrate-responsive-element-binding-protein (ChREBP), liver-X-receptors (LXRs), and peroxisome-proliferator-activated-receptors (PPARs). In addition, the patatin-like-phospholipase-domain-containing-3 (PNPLA3), a triacylglycerol-lipase, has been shown to contribute to the control of energy expenditure or storage; PNPLA3 expression is regulated by nutritional sources, especially carbohydrates (Bruschi et al., 2017). Furthermore, the mechanistic-target-of-rapamycin (mTOR), which coordinates cell growth at the organismal level, is implicated in metabolic-related disease (Saxton and Sabatini, 2017; Ji et al., 2021). Other studies have demonstrated that epigenetic processes have been linked to liver diseases, but also that the genetic profile of an individual is directly correlated to the severity of the disease, since different alleles encode metabolic enzymes with variable functional activities (Anstee et al., 2019; Ramos et al., 2021). These findings open unexplored fields in the search for innovative therapeutics, including oligonucleotide usage to reduce through RNA-interference (RNAi) the levels of molecules relevant for liver homeostasis. In this context, translational research provides a safe way to test innovative drugs at a large scale using animal models. Rodents have long been used in preclinical trials, despite the time-consuming analyses, while unpredictable side-effects of the drugs in humans cannot be excluded (Bryda, 2013; Van Norman, 2019). Thus, alternative models provide a golden solution, considering that many societies limit animal experimentation to protect them against cruelty (Doke and Dhawale, 2015; Freires et al., 2017).

## The Fruit-Fly Model

In this paradigm, the fruit-fly has emerged as a powerful system to study pathological conditions. The *Drosophila* life-cycle comprises four developmental stages: embryonic, larval, pupal, and adult. Development from embryo to adult takes about 10 days and adult lifespan 1–2 months (Johnson and Stolzing, 2019), allowing metabolic investigations on hundreds of offspring either at juvenile (larva and pupa) or adult stages. Given its powerful genetics, the *Drosophila* model has proved an efficient strategy for the study of several types of human pathologies, including metabolic (Perrimon et al., 2016; Musselman and Kuhnlein, 2018; Baenas and Wagner, 2019), neurological (Coll-Tane et al., 2019; Link and Bellen, 2020; Mariano et al., 2020; Salazar et al., 2020), cardiac (Birse et al., 2010; Piazza and Wessells, 2011; Diop et al., 2015; Guida et al., 2019), digestive (Musselman and Kuhnlein, 2018; Nayak and Mishra, 2019), and nephrocytic (Millet-Boureima et al., 2018; Rani and Gautam, 2018) comorbidities. In addition, tumor models may be induced in larvae or adult flies by genome manipulation (Pagliarini and Xu, 2003; Igaki et al., 2006; Dong et al., 2007; Gonzalez, 2013; Hirabayashi et al., 2013; Samji et al., 2021). The fruit-fly enables functional large-scale analysis to validate relevant molecules and biomarkers in a faster way than with rodent-based studies (Fernandez-Hernandez et al., 2016; Richardson and Portela, 2018; Bossen et al., 2019; Papanikolopoulou et al., 2019; Bangi, 2020).

Most of the genes and metabolic routes involved in human hepatic diseases are conserved in *Drosophila* (Table 1). The conservation between fly and mammalian genes is of utmost interest for translational studies aiming for a deeper understanding of cellular dysfunctions for which, investigation is technically restricted in mammalian models and impossible in humans.

**TABLE 1:** *Drosophila* homologs of human gene products involved in metabolic and liver disease.

**Human**	***Drosophila***	**Function in *Drosophila***	**References**
ChREBP MondoA	Mondo	Regulates sugar usage	Havula et al., 2013
SREBPs	SREBP	Regulates genes of FA synthesis	Kunte et al., 2006
LXRs	EcR	Receptor for the steroid hormone ecdysone	Parvy et al., 2014
PPARs	Eip75B	Intermediate in steroid hormone signaling	Parvy et al., 2014
PNPLA3	Brummer	Performs TAGs breakdown	Gronke et al., 2005
Perilipins	Lsd1 Lsd2	Envelop LDs	Bi et al., 2012
Apolipoproteins	apoLpp apoLTP MTP	Performs lipid transport throughout hemolymph Performs lipid loading on Lpp in enterocytes Performs lipid loading on Lpp in FB cells	Palm et al., 2012
mTOR	TOR	Regulates growth in response to nutrients	Oldham et al., 2000
Insulin IGF1 IGF2	DILPs	Regulate growth and metabolism	Brogiolo et al., 2001
IGFBP	Imp-L2	Modulates DILP activity	Honegger et al., 2008
InR IGF-R	InR	Receptor for the DILPs	Brogiolo et al., 2001
IRS1	Chico	Regulates body growth and aging	Bohni et al., 1999
Glucagon	AKH	Regulates dietary sugar response	Kim and Rulifson, 2004
Leptin	Unpaired2	As for leptin, Udp2 is a ligand for the receptor of the JAK/STAT pathway	Rajan and Perrimon, 2012
ACC1 ACC2	ACC	Rate-limiting enzyme for fatty acid synthesis	Parvy et al., 2012
FASN	FASN1 FASN2 FASN3	Ubiquitous FA-synthesis Oenocyte-restricted FA-synthesis Oenocyte-restricted FA-synthesis	Garrido et al., 2015

**FIGURE 1:**
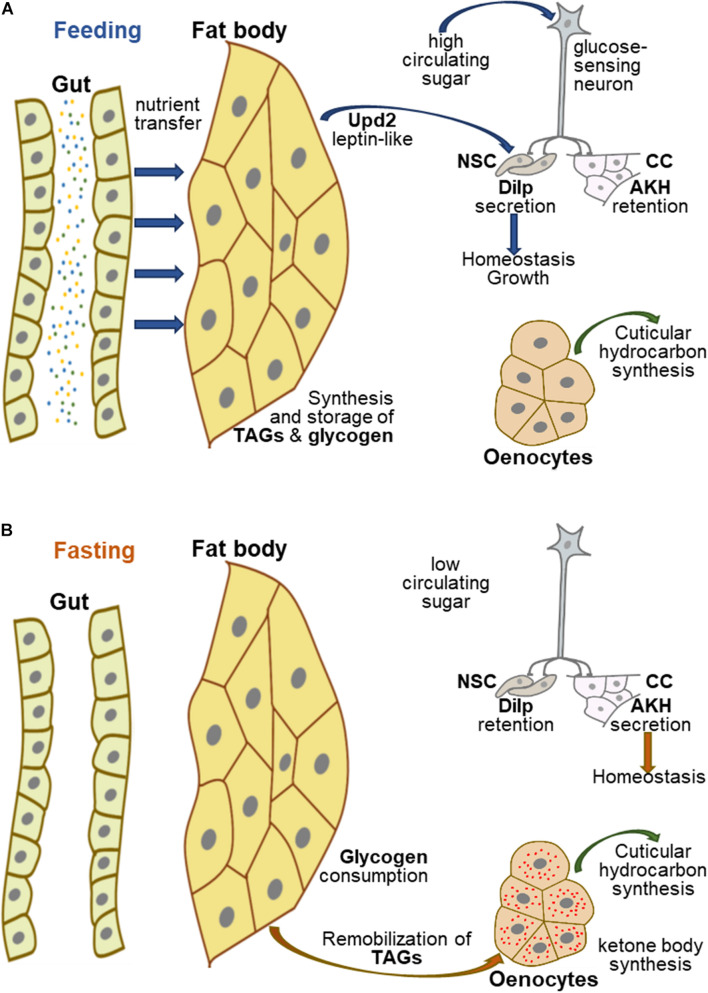
Hepatic-like homeostasis in *Drosophila*. **(A)** In fed *Drosophila*, nutrients are transferred to the appropriate organs, in particular the FB, which acts as a nutrient sensor to coordinate organismal growth and homeostasis. The FB synthesizes and stores glycogen and TAGs, and secretes several proteins, including Upd2, which can be replaced experimentally by mammalian leptin; Upd2 and glucose-sensing neurons contribute to regulating the secretion of Dilps and AKH to control growth and homeostasis. **(B)** In fasting *Drosophila*, FB glycogen and TAGs are remobilized, the latter being transferred to the oenocytes to produce ketone bodies, while glucose sensing neurons potentiate AKH secretion and Dilp retention in neurosecretory cells to maintain homeostasis.

In contrast to mammals, most invertebrates do not contain an organ equivalent to the liver, although hepatic functions are conserved. In insects, the fat body (FB) has long been considered as the liver counterpart (Li et al., 2019), although recent studies suggest that the oenocytes also accomplish hepatic-related functions (Gutierrez et al., 2007; Storelli et al., 2019). In *Drosophila*, the FB is an organ that spreads throughout the entire organism and oenocytes are groups of cells located underneath the abdominal external cuticle. In humans, dietary nutrients are transferred to the liver through a portal system and lipids are transported through the lymph stream as chylomicrons. *Drosophila* has an open circulatory system, so that nutrients crossing the intestinal epithelium enter the haemolymph, and thus, the body-wide distribution of the FB favors nutrient uptake (Figure 1A). Further, given its eating sources (mostly rotting fruits), the fruit-fly needs a powerful detoxification system that involves the gut, the FB and the oenocytes (Yang et al., 2007; Iredale, 2010; Huang et al., 2019). Over the past two decades, several studies on metabolic hepatic dysfunctions have taken advantage of the fly model (Hader et al., 2003; Villanueva et al., 2019; Ghosh et al., 2020; Hofbauer et al., 2020; Liao et al., 2020) to describe the functional activities of molecules relevant for human pathologies (Perrimon et al., 2016; Ugur et al., 2016; Heier and Kuhnlein, 2018; Hmeljak and Justice, 2019).

## Homeostatic Dysfunctions in Non-Alcoholic Fatty Liver Disease

Type 2 diabetes mellitus (T2DM) is another metabolic-related disease progressing exponentially. The T2DM hallmark is the development of insulin resistance, due to impaired response to the hormone or its reduced production by pancreatic β-cells (Boland et al., 2017; Li et al., 2021), thereby resulting in high glucose levels, increased general oxidative stress, vascular problems, and serious secondary perturbations in the physiology and the metabolism of the body (Chatterjee et al., 2017; Galicia-Garcia et al., 2020). In diabetic patients, lipid metabolism is modified, and the liver may accumulate fatty acids (FAs), which facilitates the establishment of NAFLD (Sanyal, 2019; Kuchay et al., 2020).

In NAFLD, the metabolism of both lipid and glucose in hepatic cells is disrupted (Shen et al., 2017; Sanyal, 2019). The accumulation of lipids in the hepatocytes results from changes in lipid uptake, *de novo* synthesis of FAs, β-oxidation and export of very-low-density-lipoproteins (VLDL) (Ipsen et al., 2018; Sanyal, 2019), which together affect the serum levels of TAG and cholesterol and may provoke instability of body homeostasis. Conversely, these changes in lipid metabolism, which are in part connected to insulin resistance in the hepatic tissue, reinforce the persistence of liver dysfunction, indicating that cellular metabolism is integrated at the organismal level (Chao et al., 2019; Sanyal, 2019). Under a normal and healthy homeostatic environment, hepatic cells use pyruvate to produce energy through the citric-acid cycle in mitochondria. During this process the exceeding energy is exported from the mitochondria as citrate to generate palmitic acid that can be used as a precursor of membrane lipids or esterified to TAGs and stored in cytosolic lipid droplets (LDs) (Currie et al., 2013; Ramos et al., 2021). These hepatic lipid stores can be remobilized through the β-oxidation pathway in response to energy demands, or exported from the liver in water-soluble VLDL particles comprising apolipoproteins, cholesterol and phospholipids (Ipsen et al., 2018). However, under conditions of metabolic dysfunction, excess LDs may accumulate in the liver and trigger the NAFLD, which favors lipotoxicity and, subsequently, the development of inflammatory processes and an increase in the levels of reactive oxygen species. Together, these perturbations provoke mitochondrial dysfunction and disease progression, leading to insulin resistance, T2DM and metabolic syndrome (Rosato et al., 2019).

Binding of insulin to its cognate receptor activates the mTOR signaling network (Yoon, 2017; Meng et al., 2021), which acts through a feedback loop to dampen the insulin response by inactivating insulin-receptor-substrate-1 (IRS1) and repressing the expression of the insulin receptor (InR) and insulin-like-growth-factor-1 (IGF1). Therefore, in the context of metabolic dysfunction, the mTOR signaling network is poorly activated and the subsequent absence of the negative feedback loop sustains an auto-amplification of the insulin resistance process. In this pathological condition, a number of regulatory proteins are synthesized by the hepatocytes to counteract homeostasis disruption, including Notch-receptor-2, insulin-like-growth-factor-binding-proteins (IGFBPs), the potassium-inwardly rectifying-channel-subfamily-J-member-11, chemokines and kinases (Tamarai et al., 2019).

The PPAR nuclear receptors that can act as lipid sensors to regulate metabolic homeostasis are potential targets for the development of drug therapy against NAFLD (Liss and Finck, 2017). The human genome encodes three PPAR members (α, β/δ, and γ), which are differentially expressed in tissues, although each of them may impinge on liver metabolism. PPARα is largely expressed in the liver where it controls several aspects of FA homeostasis; it is frequently reduced in patients with steatosis. PPARβ/γ is mainly expressed in muscle cells and but at lower levels in adipocytes and hepatocytes. PPARγ, which is mainly expressed in adipose tissue, is upregulated in the NAFLD pathological condition to dampen dysfunction of lipid and glucose metabolism. However, these unbalanced production of PPARs may contribute to insulin resistance and metabolic syndrome (Liss and Finck, 2017; Corrales et al., 2018), supporting the use of PPAR agonists to treat NAFLD (Seo et al., 2008; Yoo et al., 2021). The utilization Glucagon-likepeptide-1 (GLP-1) receptor agonists is another therapeutic strategy currently explored in clinical trials (Dai et al., 2020; Lv et al., 2020). GLP-1 is secreted by enteroendocrine cells after meal ingestion to potentiate insulin secretion and suppress glucagon production. The secreted bioactive forms act systemically, since the GLP-1 receptor (GLP-1R) is expressed in a number of tissues (Dai et al., 2020). The utilization of GLP-1R agonists exhibiting a longer half-life compare to that of the genuine hormone has been approved for T2DM treatment (Andersen et al., 2018). These agonists have positive effects on steatogenesis in T2DM patients (Teshome et al., 2020), but controversial results on their benefits for NAFLD have as yet restrained their use (Lv et al., 2020). Finally, a number of molecules secreted by the adipose tissue may affect inflammatory processes, insulin resistance and NAFLD progression. Adiponectin is one such factor that is down-regulated in response to hepatic stress and constitutes a promising target to treat steatosis, since studies demonstrated that increased levels of this adipokine ameliorate NAFLD (Scherer et al., 1995; Shabalala et al., 2020). Given the diversity of signals that may induce metabolic deregulations in hepatic cells, genetic investigations using alternative *in vivo* models are needed to decipher the organ interconnections that elicit the deleterious condition of NAFLD.

## Homeostasis and Metabolism in *Drosophila*

In *Drosophila*, the FB synthesizes and stores glycogen and TAG-containing LDs (Figure 1A; Gronke et al., 2003; Garrido et al., 2015; Yamada et al., 2018). In the gut, dietary lipids are hydrolyzed to glycerol, free FAs and monoacylglycerols and taken up into the enterocytes through a molecular mechanism poorly characterized as yet (Miguel-Aliaga et al., 2018; Holtof et al., 2019; Toprak et al., 2020). In the enterocytes, these molecules are converted to diacylglycerols that are loaded in the haemolymph on apolipophorin particles, functioning as lipid vehicles to the FB (Palm et al., 2012). However, FB cells deficient for fatty-acid-synthase fail to accumulate LDs, indicating that *de novo* FA synthesis is essential for lipid storage in these cells (Garrido et al., 2015). Conversely, fasting induces mobilization and consumption of lipid stores from the FB. The perilipins Lsd1 and Lsd2 envelop LDs and protect them from lipolysis, whereas the lipase, Brummer, catalyzes TAG hydrolysis (Gronke et al., 2005, 2007; Bi et al., 2012). Concurrent to fasting-induced lipid hydrolysis, oenocytes accumulate LDs (Figure 1B), suggesting that remobilized lipid stores efflux from the FB and are taken up and oxidized in the oenocytes (Gutierrez et al., 2007). This process is similar to the remobilization of lipid stores from adipocytes to hepatocytes induced by fasting in mammals (Iredale, 2010; Yamada et al., 2020). Congruently, enzymes responsible for ketone body biogenesis are highly expressed in oenocytes (Huang et al., 2019), although it has not been formally demonstrated whether other tissues (FB, muscles) may also perform β-oxidation to supply energy demand (Parvy et al., 2012). This energy mobilization process is regulated by target-of-rapamycin (TOR), which is present in two distinct complexes, TORC1 and TORC2; the former directly responds to nutrients, whereas the latter is a component of the insulin signaling pathway. The intermediates of this signaling network are conserved in the fruit-fly (Table 1), although the TORC1 and insulin/TORC2 signaling branches can work independently in most *Drosophila* tissues, including the FB (Devilliers et al., 2021).

The FB also acts as a nutrient sensor to coordinate overall body growth and homeostasis through the production of insulin-like-peptides (Dilps) by a cluster of neurosecretory cells (NSCs) (Colombani et al., 2003). Moreover, similar to mammals, sugar metabolism in flies is modulated by an insulin/glucagon-like axis (Figures 1A,B; Oh et al., 2019). The *Drosophila* genome encodes eight Dilps (Brogiolo et al., 2001), Dilp2 and Dilp5, which are produced in the NSCs, are the major regulators of sugar homeostasis (Rulifson et al., 2002). Genetic ablation of the NSCs results in an overall reduced body size and in an increase in the levels of glucose and trehalose (disaccharide of glucose) that is the main circulating sugar in insects. This phenotype resembles the metabolic defects provoked by insulin deficiency in Type-I-diabetic patients. Conversely, the *Drosophila* adipokinetic hormone (AKH) produced by the neuroendocrine corpora cardiaca (CC) controls circulating sugar levels in a glucagon-like manner (Kim and Rulifson, 2004). Both NSCs and CC secrete their products into the haemolymph, close to the *Drosophila* heart equivalent, thereby favoring hormone distribution throughout the entire body. Secretion of AKH in *Drosophila* by the CC depends on an ATP-K + -dependent channel that directly responds to circulating sugar levels (Kim and Rulifson, 2004). A pair of glucose-sensing neurons plays a pivotal role in coordinating NSC and CC functions by activating Dilp secretion, while inhibiting AKH secretion (Figures 1A,B; Oh et al., 2019). Furthermore, secretion of Dilps by the NSCs strongly relies on FB messengers that relay nutritional cues (Colombani et al., 2003; Delanoue et al., 2016). The JAK-STAT ligand Unpaired2, is one such messenger, which can be functionally replaced using molecular genetic tools by human leptin, showing that as in mammals, fat cells produce an hormone in response to nutrient load to control feeding physiology (Rajan and Perrimon, 2012). Therefore, although the mammalian liver equivalent is not a discrete organ in *Drosophila*, hepatic functions and dysfunctions are closely conserved: the FB appears to be in charge of hepatic functions related to feeding, whereas the oenocytes accomplish functions related to fasting. Further, both in mammals and flies, integration of the TOR signaling network at the organismal level is central in controlling lipid and sugar metabolism in response to the nutritional status (Schmitt et al., 2015; Sanguesa et al., 2019).

Most of the mammalian transcriptional regulators involved in NAFLD are conserved in *Drosophila* (Table 1). Mondo, the ChREBP/MondoA homolog, regulates several metabolic routes in response to dietary sugar (Mattila et al., 2015). The *Drosophila* SREBP regulates the expression of genes required for FA synthesis (Kunte et al., 2006). However, consistent with insect sterol auxotrophy (Clark and Block, 1959), SREBP activity is not regulated by sterols, but by phosphatidylethanolamine (Dobrosotskaya et al., 2002). The best homologs of LXRs and PPARs are the Ecdysone receptor (EcR) and the nuclear receptor Eip75B, respectively (Parvy et al., 2014). Ecdysone is a steroid hormone that controls developmental transitions, whereas Eip75B is an intermediate of the ecdysone signaling, whose activity depends on nitric-oxide-synthase. Surprisingly, manipulating this signaling pathway in the ecdysone-producing gland, results in a dramatic changes in LD accumulation in FB cells, indicating that this signaling pathway also impinge on lipid homeostasis (Caceres et al., 2011). In summary, the metabolic routes and most of the regulatory genes that play a critical role in NAFLD are conserved in *Drosophila*. The activities of some regulatory gene products varies as compared to that of their mammalian counterparts, but remain connected to basal metabolism. Importantly, the phenotypes induced by loss-of-function of these genes can be used as reference criteria to monitor the efficiency and the adverse effects of drug compounds in preclinical trials.

## Perspectives

Alternative models have largely contributed to our understanding of biological processes. Recent studies have shed light on potential targets for drug therapy, which need physiological validation prior to clinical trials. Thanks to a plethora of genetics tools to direct in a tissue-specific manner either over-expression or RNAi-inactivation of a gene of interest (Ugur et al., 2016; Senturk and Bellen, 2018), the fruit-fly model emerges as a powerful alternative for large-scale analyses. Collections of transgenic lines targeting a vast majority of the Drosophila genes, with a particular focus on the orthologs of disease-related-human genes, are available from Stock centers (Dietzl et al., 2007; Heigwer et al., 2018). This approach validated the functions of relevant proteins, such as CDGSH-iron-sulfur-domain-containing-protein-2, whose haplo-insufficiency causes NAFLD and promotes HCC development (Shen et al., 2017). This approach also identified genes that contribute to fat deposition (Pospisilik et al., 2010; Hoffmann et al., 2013) and whose dysregulation in humans can lead to obesity, diabetes, and NAFLD (Graham and Pick, 2017). Liver diseases are a burden in modern societies, especially NAFLD and HCC; *in vivo* investigations using *Drosophila* in translational approaches will be useful to validate enzymes and other molecules crucial for body homeostasis, increasing the chance to develop innovative therapeutic strategies.

## Author Contributions

KCMM and JM contributed equally to the content of the work. Both authors contributed to the article and approved the submitted version.

## Conflict of Interest

The authors declare that the research was conducted in the absence of any commercial or financial relationships that could be construed as a potential conflict of interest.

## Publisher’s Note

All claims expressed in this article are solely those of the authors and do not necessarily represent those of their affiliated organizations, or those of the publisher, the editors and the reviewers. Any product that may be evaluated in this article, or claim that may be made by its manufacturer, is not guaranteed or endorsed by the publisher.
